# Understanding the Potential Role of Nanotechnology in Liver Fibrosis: A Paradigm in Therapeutics

**DOI:** 10.3390/molecules28062811

**Published:** 2023-03-20

**Authors:** Sukhbir Singh, Neelam Sharma, Saurabh Shukla, Tapan Behl, Sumeet Gupta, Md. Khalid Anwer, Celia Vargas-De-La-Cruz, Simona Gabriela Bungau, Cristina Brisc

**Affiliations:** 1 Department of Pharmaceutics, MM College of Pharmacy, Maharishi Markandeshwar (Deemed to be University), Mullana-Ambala 133207, Haryana, India; 2 Chitkara College of Pharmacy, Chitkara University, Punjab 140401, India; 3 School of Health Sciences &Technology, University of Petroleum and Energy Studies, Dehradun 248007, Uttarakhand, India; 4 Department of Pharmacology, MM College of Pharmacy, Maharishi Markandeshwar (Deemed to be University), Mullana-Ambala 133207, Haryana, India; 5 Department of Pharmaceutics, College of Pharmacy, Prince Sattam Bin Abdulaziz University, Alkharj 11942, Saudi Arabia; 6 Department of Pharmacology, Bromatology and Toxicology, Faculty of Pharmacy and Biochemistry, Universidad Nacional Mayor de San Marcos, Lima 150001, Peru; 7 E-Health Research Center, Universidad de Ciencias y Humanidades, Lima 15001, Peru; 8 Department of Pharmacy, Faculty of Medicine and Pharmacy, University of Oradea, 410028 Oradea, Romania; 9 Doctoral School of Biomedical Sciences, University of Oradea, 410087 Oradea, Romania; 10 Department of Medical Disciplines, Faculty of Medicine and Pharmacy, University of Oradea, 410073 Oradea, Romania

**Keywords:** liver fibrosis, hepatic stellate cells, nanotechnology, chronic liver disease, antifibrotic activity, phytoconstituents

## Abstract

The liver is a vital organ that plays a crucial role in the physiological operation of the human body. The liver controls the body’s detoxification processes as well as the storage and breakdown of red blood cells, plasma protein and hormone production, and red blood cell destruction; therefore, it is vulnerable to their harmful effects, making it more prone to illness. The most frequent complications of chronic liver conditions include cirrhosis, fatty liver, liver fibrosis, hepatitis, and illnesses brought on by alcohol and drugs. Hepatic fibrosis involves the activation of hepatic stellate cells to cause persistent liver damage through the accumulation of cytosolic matrix proteins. The purpose of this review is to educate a concise discussion of the epidemiology of chronic liver disease, the pathogenesis and pathophysiology of liver fibrosis, the symptoms of liver fibrosis progression and regression, the clinical evaluation of liver fibrosis and the research into nanotechnology-based synthetic and herbal treatments for the liver fibrosis is summarized in this article. The herbal remedies summarized in this review article include epigallocathechin-3-gallate, silymarin, oxymatrine, curcumin, tetrandrine, glycyrrhetinic acid, salvianolic acid, plumbagin, *Scutellaria baicalnsis* Georgi, astragalosides, hawthorn extract, and andrographolides.

## 1. Introduction

The complex internal structure of the human body is made up of several intricate and sophisticated organs, each of which serves a specific purpose that forms the basis for essential physiological functions. The liver, which weighs around 1.5 kg and is located on the top side of the stomach, just behind the diaphragm, is the most important organ in the body’s gastrointestinal system [1,2]. The liver controls the production of glycogen, the breakdown of red blood cells, plasma concentrations of chemical messengers, and the elimination of toxic chemicals from the individual’s body [3]. The liver has the capacity to naturally detoxify a variety of harmful chemicals, but due to its unfavorable side effects, the liver is more vulnerable to disease. Hepatic fibrosis, end-stage liver disease, cirrhosis, portal hypertension, and hepatocellular carcinoma are all potential consequences of chronic liver diseases (CLDs), which are major reasons for morbidity, mortality, and medical expense [4]. The fibrosis represents the initial sign of liver scarring before liver cirrhosis manifests itself. Liver cirrhosis is caused by untreated excessive use of alcohol, hepatitis B, hepatitis C, hepatitis D, primary bile cirrhosis, as well as autoimmune hepatitis. There are many underlined factors behind the occurrence of this chronic disease, such as obesity, which is linked to non-alcoholic fatty liver disease. Cirrhosis is a multifactorial disease marked by tissue fibrosis/fibrotic scarring and the transformation of healthy liver anatomical structures into structurally irregular units [5]. Diffuse fibrosis, regenerative nodules, and altered lobular anatomy are all key morphological characteristics of cirrhosis [6]. The surface of the vascularized fibrotic septa determines the vascular shunts, which are an important characteristic of cirrhosis [7,8,9]. These changes cause the onset of threshold hypertension and its accompanying obstructions. Actually, the prime cause of death in cirrhotic patients is specifically portal hypertension [10].

This review discusses chronic liver disease epidemiology, the pathogenesis and pathophysiology of liver fibrosis, the signs of liver fibrosis progression and regression, the clinical assessment of liver fibrosis, the investigation of synthetic therapeutic agents for the treatment of liver fibrosis, and nanotechnology’s contribution to liver fibrosis therapy. The potential prospects for using herbal remedies include epigallocathechin-3-gallate, silymarin, oxymatrine, curcumin, tetrandrine, glycyrrhetinic acid, salvianolic acid, plumbagin, *Scutellaria baicalensis* Georgi, astragalosides, hawthorn extract and andrographolides to treat liver fibrosis are outlined in this review. The literature review was conducted using Google Scholar, PubMed, and ScienceDirect databases published in peer-reviewed journals between the years 2000 and 2023.

## 2. Epidemiology of Chronic Liver Diseases

A death rate of over 2 million per year and an affected population of over 800 million make chronic liver disorders a global crisis and pose a serious threat to global well-being. Based on the specific etiology, geographic location, and probably other variables, the overall assessed rate and pervasiveness of CLDs greatly varies in terms of sex, race, and financial status [11,12,13]. Every year, cirrhosis causes about 1,000,000 fatalities worldwide and 170,000 deaths in Europe. Since more than 5000 cirrhotic individuals are diagnosed each year in numerous European countries, therefore, cirrhosis of the liver is the primary rationale for liver transplantation [14]. Hepatocellular carcinoma (HCC) is the third leading cause of disease-related death worldwide and the fifth most common hazardous tumor, accounting for 75–80% of crucial liver-related illnesses. Hepatic cirrhosis is a key risk factor for developing HCC (approximately 700,000 cases each year, out of which 50,000 are in Europe only) [15]. There have been numerous examples investigated in non-cirrhotic persons, which revealed that progressive non-alcoholic steatohepatitis is quickly becoming prevalent throughout the world [16,17].

## 3. Pathogenesis and Pathophysiology of Liver Fibrosis

There are the following two stages of liver fibrosis activation: initiation and persistence. Several cytokines and growth factors are responsible for the initiation of hepatic fibrogenesis. The liver’s kupffer cells, hepatocytes, and endothelial cells express various isoforms of transforming growth factor beta (TGF-β), which is a major contributing factor to human fibrogenesis [18]. TGF-β1 exists as an inactive protein, and when charged with Smad protein, which is composed of structurally similar proteins, these transmit through their receptors to boost key genes, including procollagen 1 and 3 [19]. It stimulates extracellular matrix (ECM) protein synthesis, delays degradation, and aids in the conversion of hepatic stellate cells (HSC) into a cell-like myofibroblast. In experimental animals, hepatic fibrosis is reduced by platelet-derived growth factor, a potent stimulator for HSC and uptake in liver fibrosis [20]. Endothelin-1 is a powerful vasoconstrictor that promotes fibrogenesis by activating type-A receptors [21]. Another important factor in hepatic fibrogenesis is the vasoactive cytokine angiotensin-II. The pro-inflammatory cytokines, cell migration, and collagen synthesis are among the fibrogenic processes that hepatic inflammation induces and promotes in active HSCs [22]. The main adipokines that damage the liver are adiponectin, leptin, and ghrelin, which are both produced by adipose tissue and stromal cells [23,24]. For the HSC to activate and for fibrosis to form, leptin is required [25]. Yet, both in vitro and in vivo, adiponectin significantly reduces hepatic fibrosis [24]. Ghrelin reduces liver fibrosis in test animals [26]. The modulation of glucose and lipid metabolism is carried out by peroxisome proliferator active receptors (PPARs), which show reduced expression in response to HSC activation [4,27]. In contrast, PPAR-γ suppresses HSCs fibrogenic activity and lessens hepatic fibrosis [28,29]. The groups of highly conserved receptors known as toll-like receptors (TLRs) assist host cells in recognizing microbial infection and can assist in the identification of pathogen-associated molecular structures. The lipopolysaccharide-induced TLR-4 activation has been found to boost chemokine secretion and make HSCs more susceptible to TGF-β activity [30]. Furthermore, TLR-4 signaling encourages the generation of fibrogenic cytokines such as tumor necrosis factor (TNF-α), IL-1, and IL-2. A number of additional indicators point to the development of hepatic fibrosis, including alpha-smooth muscle actin (α-SMA), a reliable marker of HSC activation that occurs before the formation of fibrous tissue and is used to diagnose the onset of liver fibrosis [31,32]. It is clear that COX-2 is involved in hepatic fibrogenesis because it is expressed by activated HSCs in culture but not by quiescent HSCs [33].

**Figure 1:**
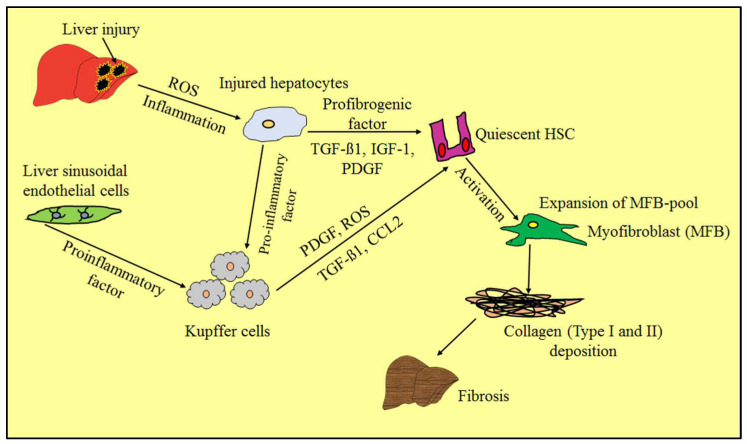
Schematic depiction of pathogenesis and pathophysiology during liver fibrosis progression. Chronic hepatic inflammation causes activation of quiescent hepatic stellate cells, which obtains transdifferentiate into MFB-like cells, which have contractile, proinflammatory, and fibrogenic properties. MFB: myofibroblast, HSC: hepatic stellate cell, ROS: reactive oxygen species, TGF-β1: transforming growth factor-β1, PDGF: platelet-derived growth factor, and CCL2: chemokine.

The progression of liver fibrosis is shown in Figure 1, along with distinct etiology and pathophysiology phases. Alterations to the liver’s architecture and increasing fibrosis are the results of long-term liver injury. Reactive oxygen species, transforming growth factor-1, platelet-derived growth factor, and chemokines created by resident Kupffer cells of the liver and dysfunctional hepatocytes cause the activation of dormant HSCs at the cellular level. These reactivated quiescent HSCs undergo additional transdifferentiation to become MFB-like cells, which are fibrogenic, pro-inflammatory, and contractile. Myofibroblasts (MFBs) play a crucial role in the generation and deposition of collagen. Numerous cell types, including localized fibroblasts, circulating fibrocytes, mesothelial cells, epithelial cells, endothelial cells, vascular smooth muscle cells, and pericytes that possess pro-fibrogenic functions tend to become articulating ECM constituents, which results in a raise in the pool of MFBs.

## 4. Evidence of Liver Fibrosis Progression and Regression

### 4.1. Liver Fibrosis Progression: Upregulation in Fibrolytic Activity

Several rat models have demonstrated the induction and quick remission of hepatic fibrosis; these results are essential for identifying the primary active mechanisms underlying cirrhosis [34,35]. Since matrix metalloproteinases (MMP) are inhibited by myofibroblast-derived TIMP-1, fibrotic ECM tends to assemble in prolonged liver damage despite ECM breakdown [36]. As the damage was stopped, TIMP-1 levels decreased, according to several studies on the treatment of hepatic fibrosis in rats [34,37]. The degradation of the ECM was caused by an increase in hepatic collagenase activity when TIMP-1 levels dropped. Several mechanistic investigations that altered TIMP to balance in situ MMP levels have proven the potent impact of these concentrations on the occurrence and treatment of liver fibrosis [38]. In terms of macrophage regeneration, it has been demonstrated that macrophages are essential for the resolution of fibrosis, highlighting their significance as mediators of effective injury healing and organ homeostasis [39]. Macrophages, which are prevalent in fibrotic liver tissue and have the propensity to induce ECM degradation and produce fibrolytic MMPs such as MMP12/13 [40,41]. The expression of TNF-related apoptosis-inducing ligands by macrophages also endorses myofibroblast apoptosis. Moreover, in hepatocellular fibroid-resolving scientific models, macrophage apoptotic cell phagocytosis results in MMP activity and accelerates ECM degradation [42]. Stabilized dendritic cells (DCs) were also examined in relation to liver fibroids treatment using transgenic CD11c diphtheria toxin receptor transgenic mice, following the use of carbon tetrachloride (CCl4)-mediated damage and in the context of adoptive transfer administered protocols, for depleting hepatic DCs during the recovery process. Moreover, DCs have demonstrated that they mediate ECM degradation, most likely through elevated MMP-9 activity [43].

### 4.2. Liver Fibrosis Regression: Cell Apoptosis or Downregulation of Hepatic Stellate Cells

It is crucial to activating HSCs in response to chronic hepatitis in the etiopathogenesis of liver cirrhosis. Recent studies in both clinical and laboratory settings have demonstrated that fibrosis can resolve once the liver injury has been treated [44]. The elimination of active HSCs through cell apoptosis or fibrolytic discharge is accurately predicted by experimental models for the recovery of fibrosis [45]. Hepatic fibrosis results in fibrous scarring that is triggered by myofibroblasts. The carbon tetrachloride liver fibrosis model causes dormant HSCs to be activated and changed into myofibroblasts. Although they had a genotype comparable to but different from quiescent HSCs, a subset of myofibroblasts during the upregulation of hepatic fibrosis was able to move at a much faster rate quickly to reactivate into myofibroblasts in comparison to fibrogenic stimuli and potentiates an adequate contribution to liver fibrosis. These myofibroblasts also resisted cell apoptosis and knockdown several hepatic fibrogenic Increased anti-apoptotic gene activity, such as that of Hspa1a/b, which helps HSCs survive in culture and in living organisms, has been linked with HSC inactivation [46].

**Figure 2:**
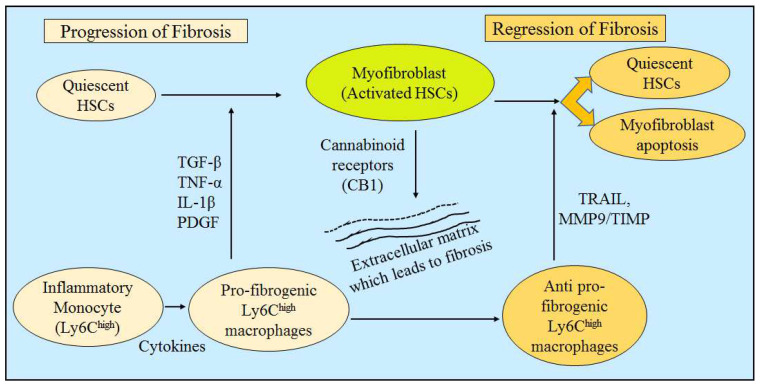
Illustrative representation indicating the interaction between hepatic stellate cells and macrophages during liver fibrosis progression and regression. HSC: hepatic stellate cell, PDGF: platelet-derived growth factor, TGF-β1: transforming growth factor-β, TNF-ɑ: tumor necrosis factor-ɑ, TRAIL: tumor necrosis (TNF)-related apoptosis-inducing ligand; TIMP: tissue inhibitors of metalloproteinases.

The stages of hepatic fibrosis advancement and regression are shown in Figure 2. When liver cells are damaged, either acutely or over time, inflammatory macrophages (Ly6C^high^) are recruited into the liver. Continuously active Ly6C^high^ macrophages act as a catalyst after chronic injury, encouraging the conversion of dormant HSCs into activated HSCs via soluble molecules such as growth factors, cytokines, and chemokines. MFBs continuously create an extracellular matrix (ECM), which causes liver scarring. If persistent damage can be stopped, such as by eliminating HCV, macrophages will change from an inflammatory to a pro-resolution Ly6C^high^ phenotype. Ly6C^high^ macrophages are unable to maintain or support the MFB phenotype. As a result, MFBs either transform back into HSCs or disappear completely due to apoptotic cell death. ECM production is stopped, and it ultimately goes away.

## 5. Clinical Evaluation of Liver Fibrosis

### 5.1. Pharmacological Elements of Liver

The development of clinical symptoms that are specific to the advanced stage of the condition and the progression of chronic liver disorders to liver cirrhosis are both largely influenced by hepatic fibrosis. Therapeutic diagnosis has been concentrated on the identification of observable conclusions regarding the mechanistic complexity of liver fibrosis development, particularly the cellular pathology assessment of hepatic tissue fibrosis. Percutaneous liver biopsy is still the gold standard for diagnosing illness, despite significant developments in imaging and laboratory testing. With collaboration and open communication with clinical data, a qualified histopathologist can also deduce a lot from a liver biopsy. The surgery is quick and painless when the patient cooperates and has a normal platelet count. The consequences, which mainly include hemorrhage but also include biliary peritonitis, hemorrhage, and perforation of other viscera, are quite uncommon in skilled hands (several major series have recorded mortality rates of between 0.01 percent and 0.1 percent) [47]. The use of ultrasonography or CT imaging can facilitate percutaneous biopsy of localized lesions. In specialized centers, there are a number of other biopsy methods that can be used on patients with aberrant clotting and/or thrombocytopenia. Except that metal coils or gelatin sponges are inserted down the digestive tract right after the biopsy, the process is exactly the same as a typical percutaneous liver biopsy. Transjugular liver biopsy is a highly helpful operation since it considerably lowers the risk of intraperitoneal bleeding in patients with severe coagulation problems. One of its limitations is a small size and “blind” collection of biopsies. However, the patient might be put to sleep and have many biopsies performed. An alternative is a laparoscopic liver biopsy, which can also be performed on a sedated patient who has moderate coagulopathy, and it has the advantage of allowing for direct liver vision.

### 5.2. Paramedical Evaluation of Liver Fibrosis

The most practical techniques for interpreting liver fibrosis include various studies based on standard laboratory criteria, specific serum biomarkers depending on the different dispersed substances compared to aggregation and remodulation of the cytosolic matrix occurring throughout the liver fibrogenic phase, and liver elastography techniques to test any scar tissues formed in the liver. These methods could help to overcome the limitations of hepatic biopsy/histochemistry and hepatic venous pressure concentration gradient evaluation, therefore obviating the necessity for invasive procedures. In addition, new test methods have gone beyond their initial goal of measuring the degree of liver fibrosis to foresee the consequences of persistent liver illness, such as portal hypertension symptoms and, in some cases, the development of HCC [48].

Non-invasive techniques are used to find the histological endpoints of severe fibrosis and cirrhosis. The significance of these endpoints depends on the histological scoring system that was employed to define them. Studies on chronic viral hepatitis that were non-invasive were validated using the METAVIR or Ishak scores. A METAVIR score of F2 or greater is considered to be significant fibrosis, whereas a METAVIR score of F4 or Ishak score of 5 has been considered to be cirrhosis. Any ongoing medical treatment and/or lifestyle changes are consequently required. Patients with cirrhosis are more prone to having significant liver disease complications and have a worse prognosis overall [49].

### 5.3. Nanotechnology-Based Diagnosis of Liver Fibrosis

The current methods for diagnosing liver fibrosis rely on intrusive techniques, which may be detrimental to patients [50]. The magnetic resonance imaging has been made available as a method for accurately diagnosing fibrosis [51]. The magnetic nanoparticles are advantageous for the detection and imaging of liver fibrosis [52]. Superparamagnetic ferric oxide nanoparticles stabilized by dextrans were used as an MRI agent to examine hepatic illness [53]. When compared to fibrotic hepatic tissue and the other half of the cytosolic matrix-rich liver parenchyma at the fibrosis level, dextran-coated superparamagnetic iron oxide nanoparticles significantly improved tissue image contrast and caused a 55 percent decrease in pixel strength. Furthermore, citrate-coated ultrasmall iron oxide nanoparticle has been investigated for MRI of liver fibrosis [54].

## 6. Synthetic Therapeutic Agents for Liver Fibrosis Management

### 6.1. Hepatic Stellate Cells-Targeting Potential Substances and Extracellular Matrix-Interfering Modulators

Promising targets for the treatment of CLDs include endocannabinoid receptors such as cannabinoid receptor type 1 (CB1) and CB2. It has been established that CB2 activation has hepatoprotective and antifibrotic properties. The selective CB2 agonist JWH-133 induces HSC cell death and quiescence by lowering the immunological response [55]. Contrarily, CB1 has the capacity to cause steatosis and encourages the deposition of the cytosolic matrix. Collagen crosslinking by members of the lysyl oxidase family, such as lysyl oxidase-like 2 (LOXL2), is a key ECM stabilizer involved in the initiation and reversal of fibrosis [56]. Several laboratory liver fibrosis models have demonstrated an antifibrotic impact from LOXL2-blocking monoclonal antibodies [57,58]. Elafibranor is a dual PPARs agonist that inhibits pro-inflammatory signaling and hepatic lipid aggregation in several NASH animal models, resulting in the treatment of liver fibrosis [59,60,61,62,63].

Renin-angiotensin system activation results in an increase in angiotensin II type 1 (AT1) receptor production, which causes activation of HSCs responsible for liver fibrosis and cirrhosis. Angiotensin II can have a profibrogenic role by inducing inflammatory reactive oxygen species, HSC proliferation, and activation, increased TGF and TIMP1 uptake, and rapid collagen sedimentation [64,65]. Therefore, angiotensin-converting enzyme (ACE) inhibitors, as well as AT1-receptor blockers, have become promising pathological treatments for liver fibrosis. The most significant inhibitor of ECM breakdown is the tissue inhibitor metalloproteinase-1 antibody, which inhibits matrix metalloproteinases. Pre-symptomatic results from carbon tetrachloride-induced hepatic fibrosis in rat models treated with anti-TIMP1 antibody revealed accumulation of fibrosis, decreased HSC activation, as well as reduced MMP2 activity [38].

### 6.2. Hepatoprotective Agents

A major factor in the emergence of CLDs is apoptosis [66]. A member of the mitogen-activated phosphokinase family, selonsertib inhibits the apoptosis-signal regulating kinase-1 (ASK1) enzyme. It is stimulated by a variety of factors, for example, TGF-β, TNF-α, or reactive oxygen species, and activation of p38/JNK signaling results in hepatic injury and fibrosis [67,68]. Pathologies of liver organ diseases demonstrate charged ASK1 signaling and blocking ASK1 in non-alcoholic steatohepatitis (NASH), a mouse model greatly reduced liver steatosis and fibrosis [68,69]. An orally active pan-caspase suppressor called “emricasan” can decrease inflammation, hepatocellular damage, and liver fibrosis in hepatic injury models [70,71].

### 6.3. Anti-Inflammatory and Antioxidant Therapies

TNF-α is a major macrophagic agent that promotes the production of pro-inflammatory markers in response to hepatic injury. A common methylxanthine derivative called pentoxifylline lowers oxidative stress while inhibiting the generation of TNF-α and several pro-inflammatory cytokines mediators [72]. Patients with NASH who received pentoxifylline treatment experienced less severe liver fibrosis [73]. An anti-inflammatory drug from the lectin family that has an important function in the treatment of liver fibrosis is called the galectin-3 antagonist. In a toxin-induced liver fibrosis murine model, the galectin-3 inhibitors galactoarabino-rhamnogalaturonan (GR-MD-02) and galactomannan (GM-CT-01) both tend to reduce the galectin-3 activity in the portal as well as septal macrophages, attenuate hepatic fibrosis, reverse hepatic cirrhosis, and reduce portal pressure [74].

The potential effectiveness of statins as cholesterol-lowering medications has been examined in a number of research studies. In rodent studies, statins improved microvascular dysfunction as well as portal hypertension in addition to the reduction in fibrosis, inflammation, and vasoprotection [75,76,77,78,79,80,81]. The recruitment of monocytes and macrophages into the liver, which results in the inflammatory response, as well as the activation of hepatic stellate cells and fibrogenesis, were all examined in another study to better understand how the interactions between chemokine receptors (types 2 and 5) and their ligands are crucial [82]. In numerous preclinical models of hepatic fibrosis, cenicriviroc, an oral antagonist in both chemokine receptor type 2 and type 5-dependent pathways, reduces the inflammatory response and fibrogenesis [83,84].

## 7. Role of Nanotechnology in Liver Fibrosis Therapy Using Synthetic Drugs

Recently, treatments based on nanotechnology have become a fascinating and superior alternative to conventional therapeutic approaches. Nanoscience is a rapidly expanding field of pharmaceutical science that focuses on the development, modification, and application of compounds in sizes range of 10–500 nanometers [85,86]. Currently, nanomedicines are made of biocompatible, sustainable materials and have a great deal of potential for targeted drug delivery. This is either performed actively by deploying tissue-or cell-specific devices that enable precise disease site targeting while minimizing side effects, or it is performed passively by increasing the physicochemical features of nano-drug carriers, such as size, shape, and surface properties [87].

Liposomes are spherical artificial vesicles that have two to three lipid bilayers around an aqueous compartment. Due to their dual structural configuration, liposomes can entrap a wide variety of both lipophilic and hydrophilic molecules. Drugs that are hydrophobic can be injected into the lipid bilayer, while those that are lipophilic can be located inside the water-soluble center of the lipid membrane. The most fundamental type of NPs are those made of liposomes, which have various advantages such as simplicity in manufacture, excellent biocompatibility, low systemic toxicity, and enhanced absorption [88,89]. Valsartan was frequently administered utilizing vitamin A-coupled liposomes. The nanomedicines enhanced the function of the hepatic Mas-receptor, and PPAR potently normalized the level of fibrogenic cytokines by increasing the permeability and effectiveness of valsartan. The platelet-derived growth factor receptor beta (PDGFR-beta) on the surface of HSCs is recognized by the cyclic peptide known as the pPB, which may also recognize vitamin A. In a study, pPB-altered liposomes were used to convert recombinant human tumor necrosis factor-related cell apoptosis-inspired ligands to the HSC surface membrane, maintaining recombinant human-TNF-related apoptosis-inspired ligands circulation in living organisms and reducing fibrosis in both vitro and vivo [90]. Likewise, CXCR4 antagonist AMD3100 has the potential to attack HSCs. To stop angiogenesis in fibrotic livers, Liu and his colleagues developed CXCR4-targeted nanoparticles to deliver siRNAs against vascular endothelial growth factor (VEGF). They discovered that a decrease in VEGF expression causes angiogenesis to be suppressed and normalizes the distorted vessels in the fibrotic livers. AMD3100-associated liposomes effectively delivered analeptic vascular endothelial growth factor siRNAs to activate chemokine receptor type 4-overexpressed HSCs. The anti-fibrotic effects of AMD3100 liposomally encapsulated were caused by the reduction of HSC activation and replication [91].

The polymeric nanoparticles are comprised of biodegradable polymers of natural origin, which include rosin, albumin, sodium alginate, gelatin, and chitosan or synthetic polymers such as polylactic acid, polylactide-co-glycolide, and polyamino acid conjugates [92,93,94,95]. According to current research, collagenase’s structure may facilitate the entry of nanocarriers into the cirrhotic liver. The self-assembled polymeric micelles based on polylactide-co-glycolide-polyspermine-polyethyleneglycol-vitamin-A polymers and loaded with two drugs, silibinin, and siCol11, effectively act in fibrotic livers and distinctively targeted over the activated HSCs and synergistically suppress collagen I production and ameliorate liver fibrosis [96]. In order to treat liver fibrosis, liver macrophages may be explored as a possible pharmacological target because they have a considerable impact on the etiology of the disease. Together with kupffer cells, liver endothelial surface cells also contain scavenger receptors that can be attacked with nanoformulations. Applications of nanoparticles in drug targeting to hepatic stellate cells or macrophages for liver fibrosis therapy are shown in Table 1.

The inorganic nanoparticles (NPs) may be employed as therapeutic agents for the therapy of liver fibrosis [105,106,107]. Type 1 collagen and alpha-smooth muscle actin activity can be suppressed by the titanium dioxide and silicon dioxide nanoparticles. Additionally, they accelerate the degradation of type 1 collagen by modifying the activity of cytosolic matrix metalloproteinases and suppressing the agonists of metalloproteinases that are found in tissues, expressing the potential anti-fibrotic properties of titanium dioxide and silicon dioxide nanoparticles inside living organisms [108]. Due to the increased antioxidant characteristics of Mn_3_O_4_ (manganese oxide) nanoparticles in stomach acid treatment, citrate-functionalized manganese oxide NPs can be employed to protect the liver against cirrhosis, fibrosis, and free oxygen radicals produced by carbon tetrachloride [109]. Zinc oxide NPs frequently reduce the lipid peroxidation levels, free oxygen radicals, and pro-inflammatory cytokines, which aid in the prevention of liver fibrosis in dimethylnitrosamine-induced liver damage [110]. In rat models of ethanol- and methamphetamine-induced hepatic damage, gold nanoparticles are reported to decrease liver cirrhosis by inhibiting the activity of stellate sinusoidal macrophages, also known as Kupffer cells [111]. According to research by Hamza et al., selenium nanoparticles and vitamin E perform synergistically to increase antioxidant enzymes, such as superoxide dismutase, catalase, and glutaredoxins, lower plasma malondialdehyde levels, and restore the normal histology and ultrastructure of hepatic tissues. These changes have been shown to have hypolipidemic and hepatoprotective effects against acrylamide-induced hepatic fibrosis in male albino mice [112].

Lipid-based nanoparticles, due to their high biocompatibility and toxic-free nature, have been regarded as the most effective vehicles [113]. Reebye et al. synthesized CEBPA-51 saRNA, which increased CEBPA mRNA levels by about 2-fold, while the non-specific control RNA oligonucleotide (siFLUC) was inactive [114]. Jimnez and his colleagues produced lipid nanoparticles and found that in animals with severe liver fibrosis, procollagen α1(I) expression was 90% suppressed, septa formation was reduced, and collagen deposition was reduced by 40–60% [115].

Protein-based nanoparticles are currently showing a lot of promise as drug-delivery vehicles for treating liver fibrosis owing to their biocompatibility and decreased immunogenicity [116]. The Cy5 labeling anti-col11 siRNA ketal cross-linked nanohydrogel particles have been demonstrated in a study to increase carrier accumulation and payload accumulation within the fibrotic tissue, limiting the progression of fibrotic fibrosis [117]. The applications of nanoparticles as drug carriers for various synthetic compounds investigated in recent years for liver fibrosis therapy are detailed in Table 2.

## 8. Emerging Perspectives of Herbal Compounds in Liver Fibrosis Therapy

In recent decades, herbal medicines comprised of biological plants and phytoconstituents have been in high demand to treat liver illnesses all over the world due to their diversity, long-lasting therapeutic benefits, and low side effects. Previous studies have demonstrated that medicinal plants and their phytoconstituents can protect the liver through a variety of processes, including the suppression of fibrogenesis, the inhibition of oxidative stress, the clearance of pathogens, and the inhibition of tumor growth [124]. When developing new hepatoprotective drugs, it is crucial to keep drug side effects to a minimum because the majority of liver injuries are chronic illnesses that require long-term treatment. Although the majority of patients think that therapeutic herbs and phytoconstituents are safe to use and have no harmful side effects, all bioactive substances, including plant drugs, have the potential to have negative consequences. More research into herbal medicines with anti-hepatic fibrotic effects is therefore necessary. The most anti-fibrotic plants are found in the families Fabaceae, Asteraceae, and Lamiaceae. These plants frequently contain phenols, quinones, glycosides, alkaloids, flavonoids, and other phytochemicals and impart significant action in the treatment of liver fibrosis.

### 8.1. Epigallocathechin-3-gallate (EGCG)

EGCG is the highly prevalent and active polyphenol found in green tea. Its effectiveness in preventing oxidative stress-related morbidities such as cancer, heart disease, and liver fibrosis has earned it prominence as a powerful antioxidant [125,126,127]. Hepatic fibrosis is caused by interstitial collagen replacement, which is promoted by increased MMP-2 activity, which is also linked to higher disruption of normal hepatic architecture [128]. Epigallocatechin-3-gallate reduced endogenous MMP-2 mRNA and protein activity. It has been demonstrated that it reduces the expression of NF-k light chain enhancer of activated B cells, which inhibits MMP-2 activity in CCl4-induced hepatic fibrosis [129]. It decreases the activity of COX-2 and inducible nitric oxide synthase by controlling the actions of C/EBP-α and NF-k light-chain enhancers of activated B-cells [130].

### 8.2. Silymarin

It is a flavonoid-rich seed extract of the *Silybum marianum*, and its principal constituents are silybin, silydianin, and silychrisin. Silybin plays an antioxidant role and promotes mitochondrial biogenesis in cirrhotic livers [131]. In the rat model of cirrhosis, silybinin can prevent citrate carrier loss and cardiolipin oxidation while also controlling oxidative stress levels in the mitochondria. Silybinin has a significant anti-inflammatory activity in a rat model of hepatic cirrhosis via boosting platelet-activating factor levels while decreasing the activity of lysophosphatidylcholine acyltransferase [132]. It helps to prevent hepatic fibrosis in CCL4-treated rats by increasing alpha-smooth muscle actin (α-SMA) levels [133]. In both rats and humans, α-SMA is a reliable biomarker of myofibroblast-like cells and a hallmark of HSC activation, which results in the deposition of fibrous tissue [134,135]. There has been a strong correlation between a decrease in α-SMA levels and a considerable reduction in the incidence of activated HSCs. As a result, silymarin helps activated HSCs to promote apoptosis [38].

### 8.3. Oxymatrine

*Sophora flavescens* Aiton (Kushen) roots have a powerful quinolizidine alkaloid called oxymatrine. For treating disorders including measles, hematochezia, diarrhea, jaundice, oliguria, and other ailments, kushen has historically been combined with another natural therapy [136]. Many studies have been conducted in recent years on its ability to prevent fibrogenesis and malignancy [137,138]. Oxymatrine reduces liver fibrosis by limiting the pro-inflammatory cytokines, interleukin-6 and TNF-α generated by carbon tetrachloride and boosting anti-inflammatory factors such as interleukin-10 [139]. It has been evident that oxymatrine controls HSCs activity by inhibiting TLR4 via the TGF-ß signaling mechanism. The liver collagen production in oxymatrine-treated rats is extensively decreased with a subsequent increase in SMAD7 and a decline of SMAD3 and cyclic adenosine monophosphate. It is linked to the control of fibrogenesis by TGF-ß, with SMAD serving as a downstream regulator [140].

Oxymatrine inhibits pro-collagen I synthesis by escalating the number of YB-1 proteins in the nucleus, which is able to mediate the regulation of the TGF-β signaling in the presence of ERK1/2 [141]. It was discovered that YB-1 expression and ERK1/2 phosphorylation level (also known as phosphorylation) were linearly associated. In a CCl4-induced fibrosis rat model, Oxymatrine caused a reduction in TIMP-1 expression [142]. It was established that the therapeutic effect of oxymatrine was increased by Arg-Gly-Asp-mediated liposome-based targeted delivery for the treatment of hepatic fibrosis by a reduction in HSC viability via triggering cell apoptosis and limiting the expression of genes involved in fibrogenesis [143].

### 8.4. Curcumin

Curcumin is a polyphenol bioactive component found in the *Curcuma longa* Linn plant that has major biological features such as anticancer, antiviral, antioxidant, and anti-inflammatory effects [144,145,146,147]. Many clinical trials have shown the impact of *Curcuma longa* on HSCs and demonstrated the promising targeted potential of curcuma on different ligands such as TGF-β [148,149], PDGFRβ, serum PDGF, CTGF, SMAD2/3 [150], tumor necrosis factor-α [151], matrix metalloproteinases, TLRs and certain inflammatory cytokines [148,149,150,151,152,153]. Curcumin suppresses the NF-kB activity in CCl4-induced liver fibrosis, and it promotes cell death through activation of caspase 3 and caspase 9, as well as the altering nuclear shape and phosphotidylserine expression [154,155]. The hepatic liver TGF-β1 activity in liver tissues is inhibited by curcumin, which prevents the aggregation of ECM in fibrosis [156]. Since the research supports that TGF-β1 activates transmembrane receptors, which in turn activate cytoplasmic proteins such as Smad proteins, which infers the nucleic information of target genes, for instance, ECM components Procollagen 1 and 3 [157].

Leptin can cause liver fibrosis, particularly in those with type 2 diabetes and obesity [158,159]. Cellular lipid deposition is reduced, and gene expression linked to lipid accumulation is downregulated when HSCs are activated. Leptin controls the activation of HSCs during fibrogenesis, which optimizes lipid metabolism and energy restoration [160,161,162]. According to research, curcumin inhibits the activation of leptin by decreasing the leptin receptor phosphorylation while enhancing PPAR-γ activity, which disrupts leptin signaling [163]. Curcumin also inhibited the effects of leptin via increasing the AMP-activated protein-kinase activity, and elevated levels of the adenosine monophosphate-activated protein kinase (AMPK) caused stimulation of lipid aggregation genes, which prevents the activation of HSCs [164].

### 8.5. Tetrandrine

Tetrandrine is an alkaloidal phytoconstituent found in *Stephania tetrandra,* which is utilized as an analgesic, antituberculosis agent, anti-inflammatory agent, anti-hypertensive agent, anti-myocardial ischemia agent, antiarrhythmic agent, anti-fibrosis agent, and anti-necrotic agent [165,166,167]. Tetrandrine combats fibrosis by preventing HSC activity and causing HSC death. By shutting down calcium ion channels, it prevents HSC activation [168,169]. It has shown that tetrandrine caused suppression in collagen deposition in the ECM in the bile duct ligation-induced liver fibrosis rat model [170]. Hsu et al. investigated the antifibrotic effects of tetrandrine and found that tetrandrine inhibited the TNF-α-induced NF-κB transcriptional activity and TGF-β1-induced alpha-SMA secretion to exert antifibrotic effects. Furthermore, tetrandrine also reduced the hepatic collagen content in the dimethylnitrosamine-induced fibrotic rat model [171]. Li et al. investigated the antifibrotic mechanism of tetrandrine alkaloid in HSC-T6 rat cell lines and found that tetrandrine exerts anti-fibrotic activity by controlling TAK1 (TGF-β-activated kinase-1), JNK (c-Jun N-terminal kinase) and NF-κB signaling pathways [172].

### 8.6. Glycyrrhetinic Acid

It is derived from *Glycyrrhiza glabra* and is thought to be a promising medication for use in clinics. In clinical trials, glycyrrhizin has demonstrated liver cirrhosis-prevention properties. It has actions that are anti-mutagenic, anti-inflammatory, antioxidant, and protective of the liver [173,174,175,176,177,178]. It is probable that glycyrrhizin’s ability to prevent cirrhosis is a result of its ability to decrease nuclear factor-kappa beta-binding activity [179]. It protects the liver from volatile OH free radicals produced from hydrogen peroxide in CCl4-induced liver fibrosis rats by upregulating Nrf-2 and boosting the expression of its target gene catalase [178]. Glycyrrhetinic acid prevents type I and III collagen expression, preventing hepatic fibrosis. Collagen I, nuclear Smad3-aggregation, and alpha-2(I) collagen genes are all eliminated in cultured HSCs [180]. Glycyrrhetinic acid therapy decreased hepatocyte apoptosis, including Bax, connective tissue growth factor, cleaved caspase-3, and HSCs activation [181].

### 8.7. Salvianolic Acid

Danshen, a Chinese herbal medicine known as *Salvia miltiorrhiza*, has a phenolic component called salvianolic acid [182]. *S. miltiorrhiza* has long been used to increase blood flow, reduce congestion, and control women’s menstrual cycles. In western Chinese medicine, it is additionally employed to treat myocardial infarction, atherosclerosis, infectious myocarditis, chronic liver disease, malignancies, and liver fibrosis [183,184,185]. Salvinolic acid’s anti-fibrotic properties are intimately related to the stimulation of HSC cell death. Salvianolic acid has the ability to suppress lipid peroxidation, reduce the activity of ALT and AST, and prevent the deposition of collagen types 1 and 3 [186]. Salvianolic acid has demonstrated therapies by delaying the onset of liver fibrosis in streptozotocin-induced diabetic rats by reducing α-SMA and TGF-β1 production. Salvianolic acid may prevent liver fibrosis brought on by bile duct ligation by triggering the SIRT1/heat shock factor 1 (HSF1) signaling pathway [187].

### 8.8. Plumbagin

Plumbagin is a napthoquinone that was produced from the herb *Plumbago zeylanica* Linn. It has a variety of therapeutic benefits, including the stimulation of apoptosis, as well as anti-inflammatory, anti-angiogenesis, antioxidant, and anti-necrosis properties [188,189]. Wei et al. investigated plumbagin’s ability to prevent liver fibrosis and found its inhibitory effect on the proliferation of HSCs [190]. According to Chen et al., plumbagin had an ameliorative effect in CCl4-induced hepatic fibrosis rats via the epidermal growth factor (EGF) receptor signaling pathway [191]. It has been shown in another study that plumbagin has anti-fibrotic action by inhibiting the NF-kβ/TLR-4 pathway, which is linked to inflammatory responses, and therefore reducing liver fibrosis [192].

### 8.9. Scutellara Baicalnsis Georgi

The active component baicalein, which is present in *Scutellaria baicalensis* Georgi, may help prevent cirrhosis and fibrosis. *S. baicalensis* extracts boosted glutathione S-transferase A5 expression in liver cells while decreasing cytochrome P450 3A2 expression. *S. baicalensis* possesses an anti-fibrosis activity, as shown by the reduction of liver malondialdehyde and hydroxyproline levels and the improvement of histological results following the use of methanolic extracts of this plant [193]. *S. baicalensis* extract also inhibits HSC activation and proliferation by causing G2/M phase cell cycle arrest and triggering ERK-dependent apoptosis through the Bax and caspases pathways [194]. In vivo tests in rats with mechanically produced scars showed that baicalein can prevent the creation of hypertrophic scars by blocking the signaling pathways of TGF-β and Smad2/3 [195]. In the CCl4-induced liver fibrosis in rats, long-term administration of baicalein decreased PDGF-beta receptor expression, which prevented HSC activation and delayed the onset of liver fibrosis [196].

### 8.10. Astragalosides

Astragalosides are known to be helpful in the treatment of liver fibrosis and are present in the plant *Astragalus mongholicus* Bunge. It caused a reduction in collagen levels, MMP-2, TIMP-2, and HSC activation and inhibited hepatic fibrosis in the CCl4 liver fibrosis rat model [197]. Guo et al. studied the in-vivo anti-fibrosis effects of astragaloside I, levistilide A, and calycosin in a dimethylnitrosamine-induced liver fibrosis model in C57BL/6 mice. They discovered that this combination significantly reduced collagen deposition, hydroxyproline content, and α-SMA expression levels in the liver tissues to exert anti-liver fibrosis effects [198]. Another research study revealed that astragalosides might prevent biliary liver fibrosis in bile duct ligation-induced liver fibrosis in rats through the inhibition of notch signaling activation [199]. Wang et al. investigated that astragaloside extracted from *Radix astragalin* inhibited the protein expression of protease-activated receptor-2 in CCl4-induced fibrotic rats to alleviate hepatic fibrosis [200].

### 8.11. Hawthorn Extract

Hamza et al. showed that Hawthorn acts as a potential carrier for the treatment of fibrosis as elucidated in research that Hawthorn modulated gene expressions of inflammatory markers such as IL-1β, TNF-α, TGF-β, NF-kB, COX-2 and reduced the myeloperoxidase activity in CCl4-induced fibrotic rats. Hawthorn’s potential effect is also linked with reduced levels of oxidative stress markers in the liver, such as malondialdehyde, and increased activity of superoxide dismutase [201].

### 8.12. Andrographolides

Lin et al. demonstrated that andrographolides have a potential role in liver fibrosis. To elucidate this role, andrographolides were administered via the intraperitoneal route for six weeks in CCl4-induced fibrotic mice. They found that andrographolides-treated CCl4-induced mice had lower levels of ALT and AST. The in-vitro research indicates that andrographolides administration can prevent liver fibrosis by blocking the TGF-1/Smad2 as well as TLR4/NF-kβ p50 pathways and attenuating the expression of profibrotic and proinflammatory proteins [202]. In order to study the antifibrotic effect of andrographolides, Yan et al. found that acetaminophen-induced liver fibrosis model in mice decreased HSC activation, decreased hepatic collagen deposition, alleviated liver oxidative stress injury, decreased production of reactive oxygen species in cells, and also enhanced nuclear factor erythroid 2–related factor 2 (Nrf2) nuclear translocation and increased the expression of Nrf2 downstream, which is a key factor for anti-fibrosis [203].

## 9. Role of Nanotechnology in Liver Fibrosis Therapy Using Herbal Compounds

Table 3 illustrates the recently explored applications of nanotechnology in the herbal-based treatment of liver cirrhosis using medicinal plant extracts and their phytoconstituents.

## 10. Conclusions and Future Perspectives

In conjunction with nanotechnology and modern medicine, herbal remedies are employed more frequently. Phytoconstituents found in abundance in plants have been shown to be helpful in the treatment of hepatic fibrosis. Numerous drugs have shown potential anti-fibrotic effects in in-vitro and in-vivo studies, but they frequently fall short of producing the desired results in clinical trials due to a lack of drug targeting in HSCs, hepatocytes, and Kupffer cells that cause the development of hepatic fibrosis. The development of nanotechnology-based antifibrotic therapy for reducing HSC activation and alleviating hepatic fibrosis in recent years has provided promising results. Currently, herbal nanoparticles are being investigated in clinical studies, and early findings suggest that they might be useful in treating fibrosis at a lower dosage. The development of nano-herbal drugs has demonstrated promise in addressing the problems of treating hepatic fibrosis while also enhancing the safety and efficacy of plant-based products.

Herbal remedies that are nanosized have the ability to enhance biological activity while also addressing some of the problems with pure herbal remedies. The development of scale-up technologies that quickly bring novel therapeutic approaches to market and the potential for gaining versatile systems that can fulfill a variety of biological and therapeutic needs provide new challenges for nanotechnology-based drug delivery systems. Since nanoparticles have the potential to have toxicological effects, a new field of toxicology called nanotoxicology has emerged, with the goal of examining the detrimental effects of nanoparticles. In the past, health innovations were assessed on their effectiveness as well as their capacity to improve patient quality of life. Currently, it is also necessary to consider healthcare costs. Although they are more expensive and structurally complex than conventional alternatives, it is anticipated that the use of nanotherapeutic materials will reduce overall healthcare spending. This decrease in healthcare expenditures is likely caused by improved nanotherapeutic effectiveness, shorter hospital stays, lower personal healthcare costs, and effective treatment for major illnesses. The market for pharmaceuticals based on nanomedicine is influenced by the pharmaceutical regulatory system, healthcare policy, demography, and general economic outlook. The companies involved in nanomedicine have developed novel strategies to meet the demands of this highly competitive industry. Nanomedicines may alter the human body in ways that are now inconceivable; therefore, it is necessary to consider both the benefits and risks of employing them.

## Figures and Tables

**Table 1:** Exploration of nanoparticles in targeted drug delivery of synthetic molecules for management of liver fibrosis.

Nanoparticle Type	Nanoparticle Subtype	Drug	Targeted Ligands	Targeted Biological Structures	Ref.
Polymeric NPs	Poly lactic co glycolic b poly(ethylene glycol) maleimide micelle	Nilotinib	Collagenase I and retinol	Hepatic stellate cell	[97]
	Diblock copolymers (POEGMA-b-VDM)	S-nitrosoglutathione	Vitamin A	Hepatic stellate cell	[98]
	Poly (lactic-co-glycolic acid) NPs	Spleen tyrosine kinase inhibitor (R406)	R406	Macrophages	[99]
	Retinol conjugated polyetherimine nanoparticle	Antisense oligonucleotide (DNA oligomers)	Retinol conjugated polyetherimine	Hepatic stellate cell	[100]
	Retinol-chitosan NPs	Atorvastatin and JQ1 (which is thienotriazolodiazepine and acts as selective inhibitor of Bromodomain-containing Protein 4 signaling pathway)	Vitamin A	Hepatic stellate cell	[101]
Inorganic NPs	Erlotinib-loaded myofibroblast-targeting nanoparticles	Erlotinib	Epidermal growth factor receptor	Hepatic sinusoids	[102]
Lipid based NPs	Liposomes	Imatinib	Vitamin A	Hepatic stellate cell	[103]
	Liposomes	Valsartan	Vitamin A	Hepatic stellate cell	[104]
	Liposomes	Antiangiogenic siRNA	Vascular endothelial growth factor siRNA (Small interfering RNA)	Hepatic stellate cell	[91]

**Table 2:** Perspectives of applications of nanoparticles as synthetic drug delivery carrier for liver fibrosis therapy.

Type of Nanoparticles (NPs)	NPs as Drug Carrier	Drug	Ref.
Lipid based NPs	RNA oligonucleotide liposomal	MTL-CEBPA (saRNA)	[114]
	Liposomes	Dexamethasone	[118]
	Cationic lipid NPs	Small interfering ribose nucleic acid to the procallogen 1 (I) gene	[115]
Inorganic NPs	Poly(ethylene glycol)-b-poly(lactic-co-glycolic acid)	Sorafenib	[119]
	Iron oxide nanoparticles	Citrate	[54]
	Calcium phosphate NPs	Tumor necrosis factor-stimulated gene 6	[120]
Polymeric NPs	Ketal cross-linked cationic nanohydrogel	Cy5-labeled anti-col1α1 siRNA	[117]
	Cationic nanohydrogel particles	Anti-col1α1 siRNA	[121]
Protein NPs	Polyavidin-based NPs	Dexamethasone	[122]
	Albumin NPs	Dexamethasone	[123]

saRNA: small activating RNA; siRNA: small interfering RNA.

**Table 3:** Breakthrough in nanotechnology-based applications of medicinal plants extracts and their bioactive compounds in therapeutics of liver fibrosis.

Phytoconstituent	Inference	Ref.
Salvianolic acid B	According to the study, SAB@MSNs-RhB had significantly greater sustained-release capability than SAB@MSNs and displayed higher release rates and concentrations in the sustained release pattern beyond 96 h. These nanoparticles also improved cellular drug uptake, bioaccessibility, higher efficacy in suppressing reactive oxygen species levels, and management of hepatic fibrosis.	[204]
Curcumin	The results of the study showed that curcumin platinum nanoparticles had greater activity at concentrations of 1.5 and 1.25 g/mL, causing the viability of NIH3T3 cells to significantly decline to 44.06% and 48.96%, respectively, for decreasing collagen production by NIH3T3 fibroblast cell line, making them a suitable candidate for hepatic fibrosis.	[205]
Phyllanthin	The results of the study demonstrated that Phyllanthin nanoparticles could reverse the biochemical and histological alterations brought on by the hepatotoxin and produced a therapeutic effect at a dose of 5 mg/kg body weight, which was half the dose of conventional medication in carbon tetrachloride (CCl4)-induced fibrotic model.	[206]
Silymarin	The study looked at the effects of silymarin-loaded Eudragit RS100 nanoparticles for hepatoprotective and anti-fibrotic benefits of silymarin in cholestatic liver fibrosis, which acts by restoring liver function through antioxidant activity, which eventually leads to enhancement in anti-fibrotic effect in bile duct ligation induced fibrotic model.	[207]
Curcumin	According to the study, the bioavailability of the drug in curcumin-loaded zein nanospheres increased by 3.24 times when compared to the free solution, making them an ideal carrier for enhanced liver targeting and anti-fibrotic effectiveness in CCl4-induced fibrotic models.	[208]
Berberine	The study demonstrated that berberine-loaded bovine serum albumin nanoparticles decreased LX-2 cell growth and showed stronger caspase 3 activations at a lower dosage in comparison to free drugs. These nanoparticles exhibited drug release of 80% in 72 h, demonstrating sustained drug release. Berberine-loaded NPs provided hepatoprotection in CCl4-induced hepatotoxicity.	[209]
San-Huang-Xie-Xin-Tang (SHXXT) decoction	The research results showed that nanoformulation of SHXXT decoction had remarkable healing potential, as evident through a reduction in AST and ALT levels in liver injury in the chloroform-induced liver fibrotic model.	[210]
Naringenin	According to the study, naringenin-loaded solid lipid nanoparticles significantly decreased CCl4-induced liver fibrosis through a decrease in biochemical markers, including serum ALP, AST, and total bilirubin, as well as pro-inflammatory markers such as TNF-, IL1β, and IL-6. In addition, pro-MMP-2 and MMP-2 activation in the HSCs were enhanced by naringenin-SLN.	[211]
Curcumin	The results of the study showed that nano-curcumin nano-chitosan mixtures had considerable hepatoprotective action through regulation of the liver enzymes ALT, AST, and ALP; AFP; caspase-3; oxidative stress biomarker such as malondialdehyde; antioxidant biomarkers such as glutathione and catalase in CCl4-induced liver fibrosis in mice model.	[212]
Hesperidin	The study’s findings demonstrated that, in contrast to hesperidin alone, DSPE-SPE-sebacic acid conjugated liposomes loaded with hesperidin provided selective targeting of HSCs in CCL4-induced rat liver fibrosis. This was demonstrated by a significant reduction in serum ALT, AST, and ALP activities as well as albumin levels.	[213]
Curcumin	In CCl4-induced liver fibrosis in rats, phosphatidylserine-modified-NLCs loaded with curcumin selectively targeted hepatic macrophages as demonstrated by their highest concentration in the liver. These nanoparticles upregulated the levels of hepatocyte growth factors and matrix metalloprotease while downregulating the amounts of collagen fibers and alpha-smooth muscle actin. They also enhanced the levels of liver enzymes and pro-inflammatory cytokines in blood circulation.	[214]

AFP: alpha-fetoprotein; ALP: alkaline phosphatase; AST: aspartate aminotransferase; ALT: alanine aminotransferase; CCl4: carbon tetrachloride; SAB@MSNs-RhB: rhodamine B covalently grafted salvianolic acid B-loaded mesoporous silica nanoparticles; DSPE: 1,2-Distearoyl-sn-glycero-3-phosphoethanolamine; NLCs: nanostructured lipid carriers.

## Data Availability

Not applicable.

## Abbreviations

α-SMA	Alpha-smooth muscle actin
ACE	Angiotensin-converting enzyme
AFP	Alpha-fetoprotein
ALP	Alkaline phosphatase
ALT	Alanine aminotransferase
API	Active pharmaceutical ingredient
ASK-1	Apoptosis-signal regulating kinase-1
AST	Aspartate aminotransferase
CAT	Catalase
CB1	Cannabinoid receptor type 1
CCL2	Chemokine
CCl4	Carbon tetrachloride
CLDs	Chronic liver diseases
COX	Cyclooxygenase
CRMs	Cis-acting regulatory modules
CTGF	Connective tissue growth factor
DCs	Dendritic cells
DSPE	1,2-Distearoyl-sn-glycero-3-phosphoethanolamine
ECM	Extracellular matrix
EGCG	Epigallocathechin-3-gallate
EGFR	Epidermal growth factor receptor
EMT	Epithelial-mesenchymal transition
GM-CT-01	Galactomannan
GR-MD-02	Galactoarabino-rhamnogalaturonan
GSH	Glutathione
HCC	Hepatocellular carcinoma
HCV	Hepatitis C virus
HSCs	Hepatic stellate cells
IL-1	Interleukins-1
IL-2	Interleukins-2
IL-13	Interleukin-13
JNK	c-Jun N-terminal kinases
LOXL2	Lysyl oxidase-like 2
LPO	Lipid peroxidation
MAPK	Mitogen-activated protein kinase
MDA	Malonaldehyde
MFBs	Myofibroblasts
MMP-1	Matrix metalloproteinase-1
MRI	Magnetic resonance imaging
NASH	Non-alcoholic steatohepatitis
NFkB	Nuclear factor kappa-light-chain-enhancer of activated B cells
NLCs	Nanostructured lipid carriers
Nrf2	Nuclear factor erythroid 2–related factor 2
OM	Oxymatrine
PPARs	Peroxisome proliferator active receptors
PDGF	Platelet-derived growth factor
PDGFR	Platelet derived growth factor receptor
PS	Phosphatidylserine
ROS	Reactive oxygen species
SAB@MSNs-RhB	Rhodamine B covalently grafted salvianolic acid B-loaded mesoporous silica nanoparticles
TGF-β	Transforming growth factor-β
TIMP-1	Tissue inhibitor metalloproteinase-1
TLRs	Toll-like receptors
TNF-ɑ	Tumor necrosis factor-ɑ
TRAIL	Tumor necrosis (TNF)-related apoptosis-inducing ligands
VEGF	Vascular endothelial growth factor
